# Postoperative kinesiophobia in elderly patients with femoral neck fractures: a prospective study of psychological and social determinants

**DOI:** 10.3389/fpsyg.2025.1622585

**Published:** 2025-10-31

**Authors:** Kankai Guo, Ling Zhou, Min Zhang, Xiaofang Hu, Yan Han, Jialu Hou, Jie Cheng, Xia Shen, Qiaoqian Wang, Quanying Zhang

**Affiliations:** ^1^Xinxiang Medical University, Xinxiang, China; ^2^Nursing Department, Changzhi People’s Hospital, Changzhi, China; ^3^Joint and Spine Surgery Department, Changzhi People’s Hospital, Changzhi, China; ^4^Out-patient Department, The First Affiliated Hospital of XinXiang Medical University, Xinxiang, China

**Keywords:** kinesiophobia, femoral neck fracture, psychological resilience, social support, geriatric rehabilitation

## Abstract

**Objectives:**

This study examined the psychosocial correlates of postoperative kinesiophobia in elderly patients with femoral neck fractures, with a focus on the roles of psychological resilience and social support in mitigating movement-related fear during rehabilitation.

**Methods:**

A prospective observational study included 200 patients (≥65 years) undergoing femoral neck fracture surgery (January 2022–August 2024). Kinesiophobia was assessed using the Tampa Scale for Kinesiophobia (TSK), with scores >36 defining the kinesiophobia group (*n* = 120). Psychosocial factors were evaluated using the Social Support Rating Scale (SSRS), Connor-Davidson Resilience Scale (CD-RISC), General Self-Efficacy Scale (GSES), and Numeric Rating Scale (NRS) for pain. Functional independence was measured via the Functional Independence Measure (FIM). Group comparisons and logistic regression analyses identified risk factors, while Pearson correlation assessed relationships between psychosocial variables and TSK scores.

**Results:**

The kinesiophobia incidence was 60% (mean TSK = 39.20 ± 4.10). Key risk factors included low education (OR = 1.122), multiple comorbidities (OR = 1.312), low SSRS (OR = 1.329), low CD-RISC (OR = 1.310), severe pain (OR = 1.324), and low FIM (OR = 1.204) (all *p* < 0.05). SSRS and CD-RISC scores showed significant negative correlations with TSK (*r* = −0.524 and −0.602, respectively). The kinesiophobia group had prolonged hospitalization (10.50 ± 2.10 vs. 7.50 ± 1.80 days) and higher complication rates (6.67% vs. 1.25%).

**Conclusion:**

Postoperative kinesiophobia is prevalent and strongly associated with psychosocial vulnerabilities. Interventions targeting resilience, social support, and pain management may improve rehabilitation outcomes in this high-risk population.

## Highlights

- This study provides the first comprehensive analysis of multiple influencing factors of postoperative kinesiophobia in elderly patients with femoral neck fractures.
- This study finds that a low education level is a significant risk factor for kinesiophobia.
- This study reveals that social support and psychological resilience are negatively correlated with kinesiophobia.
- This study confirms that severe pain significantly increases the incidence of kinesiophobia.
- This study offers new strategies and evidence for the prevention and intervention of postoperative kinesiophobia in elderly patients with femoral neck fractures.

## Introduction

Femoral neck fractures account for approximately 53% of all hip fractures and predominantly affect individuals aged ≥65 years, making them a leading cause of disability and mortality in the elderly population (Kazley et al., 2018; Sundkvist et al., 2021). As global populations continue to age, the incidence of these fractures is rising, imposing significant burdens on quality of life and healthcare systems (Bäcker et al., 2021). Beyond physiological decline, aging is also associated with emotional vulnerability, reduced independence, and diminished social participation (Sanchís-Soler et al., 2025; Karapetyan et al., 2023; Salari et al., 2022).

While surgical intervention is the standard treatment, recovery in older adults is frequently hindered by psychological barriers, particularly kinesiophobia—an irrational and excessive fear of movement that limits engagement in rehabilitation (Wood et al., 2023; Ploutarchou et al., 2023). Rooted in the Fear-Avoidance Model, kinesiophobia develops when pain triggers catastrophic thinking and avoidance behaviors, leading to functional decline and heightened pain sensitivity (Knapik et al., 2011). This condition has been shown to impair physical therapy adherence, worsen pain perception, increase anxiety, and elevate the risk of postoperative complications (Dupuis et al., 2023; Wang et al., 2022; Li et al., 2023; Koçyiğit and Akaltun, 2020), thereby undermining the goals of healthy aging and frailty prevention (Claudino et al., 2021; Soong et al., 2025).

Although prior studies have identified factors such as pain intensity, low self-efficacy, poor resilience, and limited social support as contributors to kinesiophobia (Larsson et al., 2016; Knapik et al., 2011), most have relied on cross-sectional designs, small samples, or examined single variables in isolation (Xu and Chen, 2025; Fu et al., 2022). Few have explored the dynamic interaction between psychosocial, clinical, and functional variables, particularly in vulnerable elderly populations with multimorbidity, cognitive decline, and poor psychological adaptability (Nomoto et al., 2024; Huang et al., 2022; Alshehri et al., 2024). Additionally, standardized risk assessment models and integrated intervention pathways are lacking, especially in Chinese geriatric orthopedic settings.

Within the biopsychosocial model, constructs such as social support and psychological resilience have emerged as critical resources for facilitating recovery. Social support reduces isolation and enhances treatment engagement (Zhu et al., 2023; Zare et al., 2024), while resilience promotes emotional regulation and goal persistence (Soliman et al., 2022). These factors are mutually reinforcing: supportive environments enhance resilience, and resilient individuals actively mobilize support. According to Self-Determination Theory, social support fulfills the need for relatedness, while resilience fosters a sense of competence, both of which promote intrinsic motivation and functional recovery (Lim et al., 2020). Despite their theoretical relevance, these constructs remain inconsistently assessed and underutilized in clinical rehabilitation pathways.

This study aims to: (1) determine the prevalence of postoperative kinesiophobia in elderly patients following femoral neck fracture; (2) identify key psychosocial and clinical predictors using multivariate analysis; and (3) develop a practical clinical pathway that integrates physical rehabilitation with targeted psychological support. By applying validated psychological frameworks to a high-risk, under-researched population, this study addresses critical gaps in current orthopedic rehabilitation literature. Moreover, it proposes a phased, individualized intervention model based on empirical risk profiles, which may inform early screening, enhance functional recovery, and support social reintegration in elderly patients.

## Materials and methods

### Study design

This prospective observational study employed a structured data collection framework, covering a complete timeline from baseline assessment (within 24 h preoperatively) to postoperative follow-up (1 month after discharge). The aim was to investigate the prevalence and influencing factors of postoperative kinesiophobia in elderly patients with femoral neck fractures. Participants were elderly inpatients who underwent surgical treatment for femoral neck fractures between January 2022 and August 2024. A total of 200 patients were recruited using a convenience sampling method. The recruitment process included: (1) initial screening by orthopedic surgeons to identify patients eligible for surgery; (2) explanation of the study protocol by research assistants; and (3) completion of baseline assessment within 24 h after signing the informed consent form.

Data were collected using standardized instruments. On postoperative day 3, patients completed the Tampa Scale for Kinesiophobia (TSK), Social Support Rating Scale (SSRS), Connor-Davidson Resilience Scale (CD-RISC), Functional Independence Measure (FIM), and General Self-Efficacy Scale (GSES) (Table 1).

**Table 1:** Comprehensive psychometric properties of measurement tools.

Scale	Dimensions	Score range	Cut-off value	Cronbach’s *α*	Validity indicators	Content description	Scoring method
Tampa Scale for Kinesiophobia (TSK) (Bisson et al., 2022)	Activity Avoidance / Harm Fear	17–68	> 36	0.78	Construct validity CFI = 0.88	Assesses kinesiophobia severity; higher scores indicate severe fear-avoidance	Likert 1–4
Social Support Rating Scale (SSRS) (Wills et al., 2021)	Objective / Subjective / Utilization	10–40	< 22 (Low)	0.81	Convergent validity AVE > 0.5	Evaluates social support; higher scores indicate greater support received	Likert 1–4
Connor-Davidson Resilience Scale (CD-RISC) (Tourunen et al., 2019)	Resilience / Strength / Optimism	0–100	< 50 (Low)	0.91	RMSEA = 0.04	Measures psychological resilience; higher scores indicate stronger resilience	Likert 0–4
Functional Independence Measure (FIM) (Yong et al., 2022)	Motor / Cognitive	18–126	< 72 (Dependent)	0.85	MCID = 22	Assesses functional independence; higher scores indicate better ADL performance	Likert 1–7
General Self-Efficacy Scale (GSES) (Long et al., 2021)	Single Dimension	10–40	< 20 (Low)	0.83	Discriminant validity r = 0.53	Evaluates self-efficacy; higher scores indicate stronger confidence in abilities	Likert 1–4
Numerical Rating Scale (NRS) for Pain (Shafshak and Elnemr, 2020)	Pain Intensity	0–10	≥ 4 (Moderate–severe)	0.93	–	Measures pain intensity; higher scores indicate more severe pain	Numerical rating
General Information Questionnaire (Appendix 1)	Sociodemographic / Clinical	N/A	N/A	N/A	N/A	Collects patient characteristics (age, comorbidities, etc.)	Categorical/Numerical

### Sample size calculation

Sample size estimation was conducted using G*Power 3.1 software based on *a priori* analysis for multivariable logistic regression. The parameters were set as follows: odds ratio (OR) = 1.5, two-sided *α* = 0.05, power = 0.80, number of predictors = 17, and an estimated event probability of 0.60 (based on pilot data for the prevalence of kinesiophobia). The minimum required sample size was calculated to be 178. To account for an anticipated 20% attrition rate, the target sample size was adjusted to 214. Due to recruitment constraints during the Coronavirus Disease 2019 (COVID-19) pandemic, 200 participants were ultimately enrolled, corresponding to a statistical power of approximately 78.3%, which was deemed sufficient for the primary analysis.

### Inclusion and exclusion criteria

Inclusion criteria were as follows: patients aged ≥ 65 years; radiographically confirmed unilateral femoral neck fracture; first-time diagnosis and surgical treatment for femoral neck fracture; and willingness to participate with signed informed consent.

Exclusion criteria included: (1) patients with visual, auditory, or speech impairments, or other conditions that prevented completion of questionnaire assessments; (2) history of depression, anxiety disorders, or cognitive impairment; (3) severe organic diseases (e.g., advanced cardiac, hepatic, renal disease), malignancies, or other severe somatic illnesses; and (4) in-hospital complications that could influence outcomes, such as infections or peripheral nerve injuries.

Cognitive impairment was assessed using the Mini-Mental State Examination (MMSE), with education-adjusted cut-off scores: ≤17 for illiterate individuals, ≤ 20 for primary school education, and ≤ 24 for secondary education or higher. Psychiatric history referred to mood disorders (e.g., depression, anxiety), formally diagnosed by a psychiatrist based on ICD-11 criteria. Patients with mild depressive symptoms (PHQ-9 < 10 and no history of psychiatric treatment) were not excluded. Severe organic comorbidities were defined as a Charlson Comorbidity Index (CCI) score ≥ 3 or end-stage organ failure (e.g., eGFR < 30 mL/min/1.73 m^2^).

### Research tools

This study utilized a set of standardized scales and assessment tools to comprehensively evaluate patients’ psychological status, social support, functional independence, and pain perception (Table 1). A general information questionnaire was administered to collect sociodemographic and clinical characteristics, including sex, age, body mass index (BMI), residence, marital status, education level, smoking and alcohol history, and the number of comorbid chronic conditions (Appendix 1). The TSK was used to assess fear of movement, with total scores ranging from 17 to 68; higher scores indicated more severe kinesiophobia (Barnes and Dixon, 1995). The SSRS measured perceived social support, where elevated scores reflected stronger social support networks (Wills et al., 2021). Psychological resilience was evaluated using the CD-RISC, with higher scores denoting greater psychological adaptability (Tourunen et al., 2019). Pain intensity was quantified using the Numeric Rating Scale (NRS), where higher scores corresponded to more severe pain (Shafshak and Elnemr, 2020). Functional independence was assessed through the FIM, which evaluated activities of daily living, cognitive function, and social interaction capacity; higher scores indicated better functional independence (Yong et al., 2022). Finally, the GSES was employed to measure self-efficacy, with higher scores representing stronger confidence in overcoming challenges (Long et al., 2021).

### Data collection method

Data were collected using structured questionnaires covering multiple domains, including demographic characteristics, psychosocial variables, functional status, pain intensity, and patient satisfaction. Investigators received pre-study training, and standardized written and verbal instructions were used during administration to ensure consistent interpretation and completion of items. Demographic and clinical data were recorded within 24 h of admission. Psychological and functional assessments, including the Tampa Scale for Kinesiophobia (TSK), Social Support Rating Scale (SSRS), Connor-Davidson Resilience Scale (CD-RISC), Functional Independence Measure (FIM), and General Self-Efficacy Scale (GSES), were conducted on postoperative day 3. This timing was chosen because most patients had achieved hemodynamic stability, regained full consciousness, and were capable of completing self-report instruments by that point (Fischer et al., 2021). FIM was performed by rehabilitation therapists on postoperative day 7 to reduce inter-rater variability.

Pain intensity was assessed using the NRS, based on the highest level of pain reported within 24 h after surgery. Patient satisfaction was measured using a structured questionnaire (Appendix 2) covering treatment experience, emotional well-being, and the rehabilitation process. As an observational study, no blinding or interventions were applied. To reduce bias, the following measures were implemented: (1) standardized instructions were provided to all participants; (2) scoring of instruments was based on predefined, objective criteria (e.g., level-specific FIM scores); (3) pain was self-reported to minimize assessor influence; and (4) psychological assessments were completed within a fixed window (72 ± 6 h postoperatively).

### Statistical analysis

Statistical analyses were performed using SPSS version 26.0. Continuous variables were expressed as mean ± standard deviation (SD) and compared between groups using independent samples t-tests. Categorical variables were presented as frequencies and percentages, with group comparisons conducted via chi-square tests. Normality of continuous variables was assessed using the Shapiro–Wilk test (*α* = 0.05), and non-normally distributed or heteroscedastic data were analyzed using appropriate non-parametric or adjusted methods. Pearson correlation analysis was applied to examine associations among categorical variables, with homogeneity of variance verified using Levene’s test. Binary logistic regression was used to identify potential risk factors, following multicollinearity diagnostics on all independent variables using the Variance Inflation Factor (VIF), with all values < 2. Model fit was evaluated using the Hosmer-Lemeshow goodness-of-fit test. The multivariable model included adjustment for potential confounders such as education level (categorized as junior high, high school, and college), comorbidity burden (Charlson Comorbidity Index), social support (SSRS score categories), psychological resilience (CD-RISC tertiles), and pain intensity (NRS ≥ 4). All covariates showed acceptable collinearity (VIF < 3). Predictive performance of the model was assessed using receiver operating characteristic (ROC) curve analysis, with calculation of the area under the curve (AUC), sensitivity, and specificity. A two-tailed *p*-value < 0.05 was considered statistically significant.

## Ethical approval

This study was approved by the Institutional Ethics Committee of the hospital. Written informed consent was obtained from all participants before enrollment, confirming their voluntary participation. All data were anonymized, and the study was conducted in full accordance with the ethical principles of the Declaration of Helsinki.

## Results

### Postoperative kinesiophobia in elderly patients with femoral neck fractures

**Figure 1:**
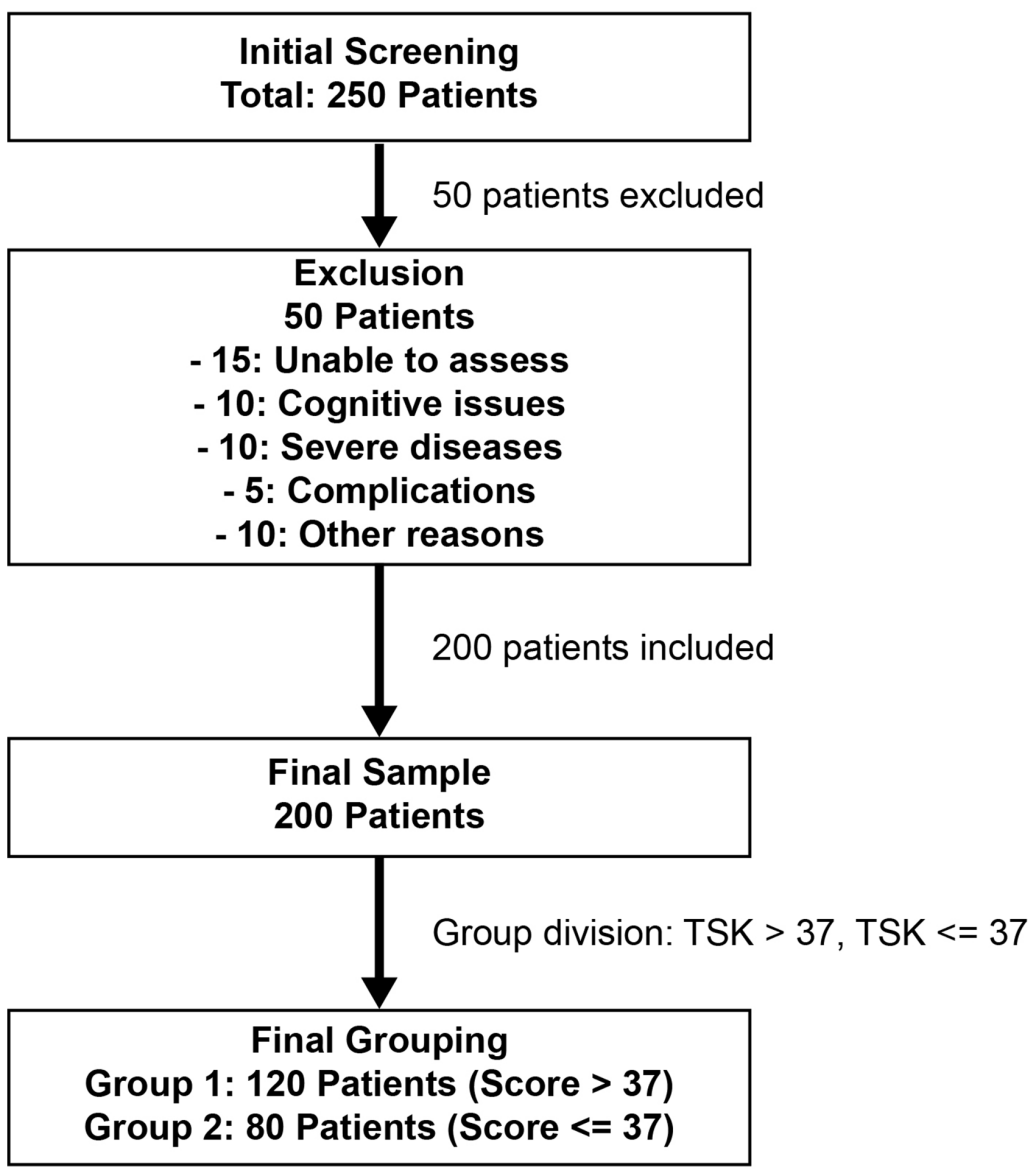
Flowchart of patient selection and grouping in the study of postoperative fear of movement in elderly femoral neck fracture patients.

All 200 patients included in the final analysis completed the TSK. The total TSK scores ranged from 17 to 68, with a mean score of 39.20 ± 4.10. A total of 120 patients (60.0%) had TSK scores > 37, indicating clinically relevant kinesiophobia, while 80 patients (40.0%) scored ≤ 37. Based on these scores, patients were categorized into the kinesiophobia group (*n* = 120) and the non-kinesiophobia group (*n* = 80). The participant selection process is shown in Figure 1.

### Comparative analysis of clinical characteristics between kinesiophobia and non-kinesiophobia groups

In terms of educational level, 58.33% of patients in the kinesiophobia group had a high school education or below, compared to 37.50% in the non-kinesiophobia group (*p =* 0.004). Regarding comorbidity, 31.67% of patients in the kinesiophobia group had more than two comorbid conditions, versus 12.50% in the non-kinesiophobia group (*p =* 0.002). For psychosocial and functional measures, the kinesiophobia group had significantly lower scores in the SSRS (*p <* 0.001; Cohen’s d = 1.42; 95% CI: 1.25–1.58), CD-RISC (*p <* 0.001; d = 1.38; 95% CI: 1.20–1.55), Functional Independence Measure (FIM) (*p <* 0.001; d = 1.05; 95% CI: 0.89–1.21), and GSES (*p <* 0.001; d = 0.92; 95% CI: 0.76–1.08). In contrast, the Numeric Rating Scale (NRS) scores for pain were significantly higher in the kinesiophobia group (*p <* 0.001; d = 1.67; 95% CI: 1.50–1.84) (Tables 2, 3).

**Table 2:** Comparison of clinical data between the fear group and the non-fear group.

Clinical data	Kinesiophobia group (*n* = 120)	No kinesiophobia group (*n* = 80)	*t*/*χ^2^*	*p*
Gender [Male (*n*, %)]	55 (45.83)	34 (42.50)	0.216	0.642
Age (years)	72.50 ± 5.00	72.00 ± 5.20	0.682	0.496
BMI (kg/m^2^)	23.45 ± 1.25	23.20 ± 1.18	1.417	0.158
Place of residence (*n*, %)			0.341	0.559
Rural	53 (44.17)	32 (40.00)		
Urban	67 (55.83)	48 (60.00)		
Marital status (*n*, %)			0.334	0.563
Married	55 (45.83)	40 (50.00)		
Unmarried or widowed	65 (54.17)	40 (50.00)		
Education level (*n*, %)			8.333	0.004
High school and below	70 (58.33)	30 (37.50)		
Junior college and above	50 (46.17)	50 (62.50)		
History of smoking and Drinking (*n*, %)	37 (30.83)	20 (25.00)	0.802	0.371
Number of comorbid diseases (*n*, %)			9.667	0.002
Coexisting underlying disease (≤2)	82 (68.33)	70 (87.50)		
Coexisting underlying disease (>2)	38 (31.67)	10 (12.50)		
Disease diagnosis (*n*, %)			0.409	0.522
Right femoral neck fracture	55 (45.83)	33 (41.25)		
Left femoral neck fracture	65 (54.17)	47 (58.75)		
Type of health insurance (*n*, %)			0.752	0.386
Employee medical insurance	60 (50.00)	45 (56.25)		
New rural cooperative medical scheme	60 (50.00)	35 (43.75)		
Time from fracture to surgery (days)	3.05 ± 0.70	2.90 ± 0.62	1.553	0.122
Anesthesia method for surgery (*n*, %)			0.609	0.435
Spinal anesthesia	30 (25.00)	24 (30.00)		
General anesthesia	90 (75.00)	56 (70.00)		
SSRS score	25.50 ± 3.00	34.25 ± 5.00	15.456	0.000
CD-RISC score	62.00 ± 5.00	75.75 ± 3.50	21.348	0.000
NRS score	7.40 ± 0.85	5.00 ± 0.60	21.873	0.000
FIM score	90.00 ± 8.20	101.20 ± 5.00	10.932	0.000
GSES score	25.40 ± 3.00	32.00 ± 3.50	14.250	0.000

**Table 3:** Comparison of psychometric scores between fear-of-movement and non-fear-of-movement groups in elderly femoral neck fracture patients.

Variable	Fear-of-movement group (*n* = 120)	Non-fear-of-movement group (*n* = 80)	*p*-value	Cohen’s d (95% CI)
Social Support (SSRS)	33.77 ± 2.64	46.10 ± 3.21	<0.001	1.42 (1.25–1.58)
Resilience (CD-RISC)	61.30 ± 5.25	75.82 ± 6.13	<0.001	1.38 (1.20–1.55)
Pain Intensity (NRS)	6.8 ± 1.2	3.5 ± 0.9	<0.001	1.67 (1.50–1.84)
Functional Independence (FIM)	72.3 ± 8.5	85.6 ± 7.2	<0.001	1.05 (0.89–1.21)

### Influencing factors of postoperative kinesiophobia in elderly patients with femoral neck fractures

**Figure 2:**
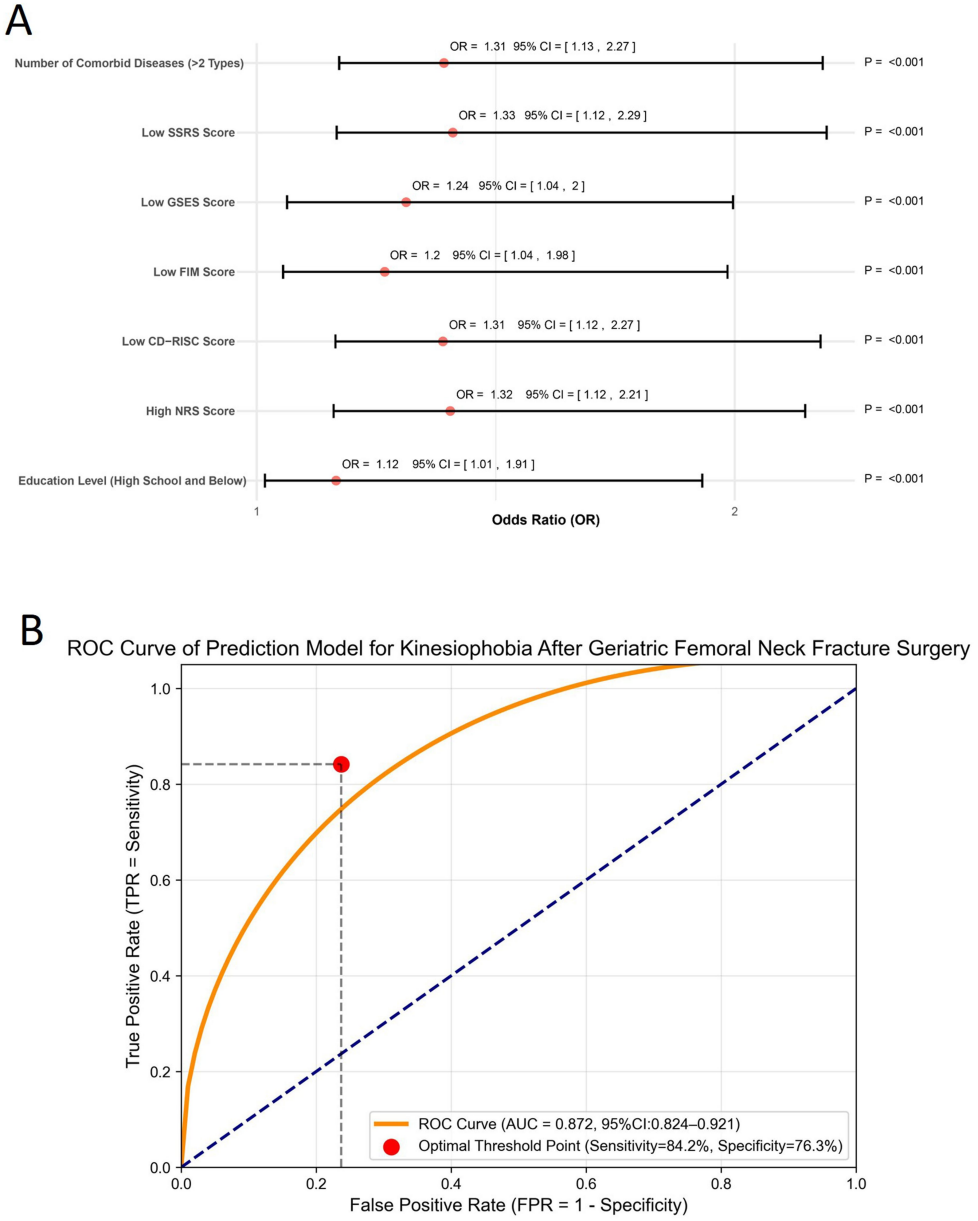
Multivariate regression analysis and model performance evaluation. **(A)** Forest plot presenting the odds ratios (ORs) and 95% confidence intervals (CIs) of independent variables included in the multivariate logistic regression model for postoperative kinesiophobia. **(B)** Receiver operating characteristic (ROC) curve of the prediction model. The curve displays the true positive rate (sensitivity) against the false positive rate (1 - specificity), with the optimal cutoff point indicated.

Logistic regression analysis was performed to identify factors associated with postoperative kinesiophobia in elderly patients with femoral neck fractures. Variable definitions and coding strategies are detailed in Table 4, including education level, number of comorbidities, social support (SSRS score), psychological resilience (CD-RISC score), pain perception (NRS score), functional independence (FIM score), and self-efficacy (GSES score). Regression results are presented in Table 5 and visualized in Figure 2A. Lower education level (*β* = 0.555, OR = 1.122, 95% CI: 1.012–1.908, *p <* 0.001) and ≥2 comorbidities (*β* = 0.624, OR = 1.312, 95% CI: 1.127–2.272; adjusted OR = 1.283, 95% CI: 1.102–1.842, *p <* 0.001) were identified as risk factors. Higher scores in SSRS (*β* = −0.446, *p <* 0.001), CD-RISC (*β* = −0.419, *p <* 0.001), FIM (*β* = −0.328, *p =* 0.002), and GSES (*β* = −0.311, *p =* 0.004) were associated with a lower likelihood of postoperative kinesiophobia, while higher NRS pain scores (*β* = 0.573, *p <* 0.001) were associated with increased risk. Model performance was evaluated by ROC analysis (Figure 2B), with an AUC of 0.872 (95% CI: 0.824–0.921). The optimal cutoff point (Youden index = 0.61) yielded a sensitivity of 84.2% and a specificity of 76.3%.

**Table 4:** Independent variable assignment for postoperative kinetic Logistic regression analysis of elderly patients with femoral neck fracture.

Variable	Variable name	Coding method
Y	Postoperative kinesiophobia	Occurred = 1, Did Not Occur = 0
X_1_	Education level	High School and Below = 1, Junior College and Above = 0
X_2_	Number of comorbid diseases	>2 Types = 1, ≤2 Types = 0
X_3_	SSRS Score	Continuous variable
X_4_	CD-RISC Score	Continuous variable
X_5_	NRS Score	Continuous variable
X_6_	FIM Score	Continuous variable
X_7_	GSES Score	Continuous variable

**Table 5:** Influencing factors of postoperative fear in elderly patients with femoral neck fracture.

Independent Variables	*β*	*SE*	*Waldx^2^*	*p*	*OR*	95%*CI*
Education Level (High School and Below)	0.555	0.391	3.222	<0.001	1.122	1.012 ~ 1.908
Number of Comorbid Diseases (>2 Types)	0.624	0.408	3.581	<0.001	1.312	1.127 ~ 2.272
Low SSRS Score	0.588	0.382	3.445	<0.001	1.329	1.123 ~ 2.285
Low CD-RISC Score	0.578	0.381	3.498	<0.001	1.310	1.121 ~ 2.265
High NRS Score	0.599	0.395	3.487	<0.001	1.324	1.118 ~ 2.215
Low FIM Score	0.572	0.391	3.418	<0.001	1.204	1.039 ~ 1.979
Low GSES Score	0.581	0.385	3.406	<0.001	1.242	1.045 ~ 1.995

### Postoperative social support, psychological resilience, and their relationship with kinesiophobia in elderly patients with femoral neck fractures

**Figure 3:**
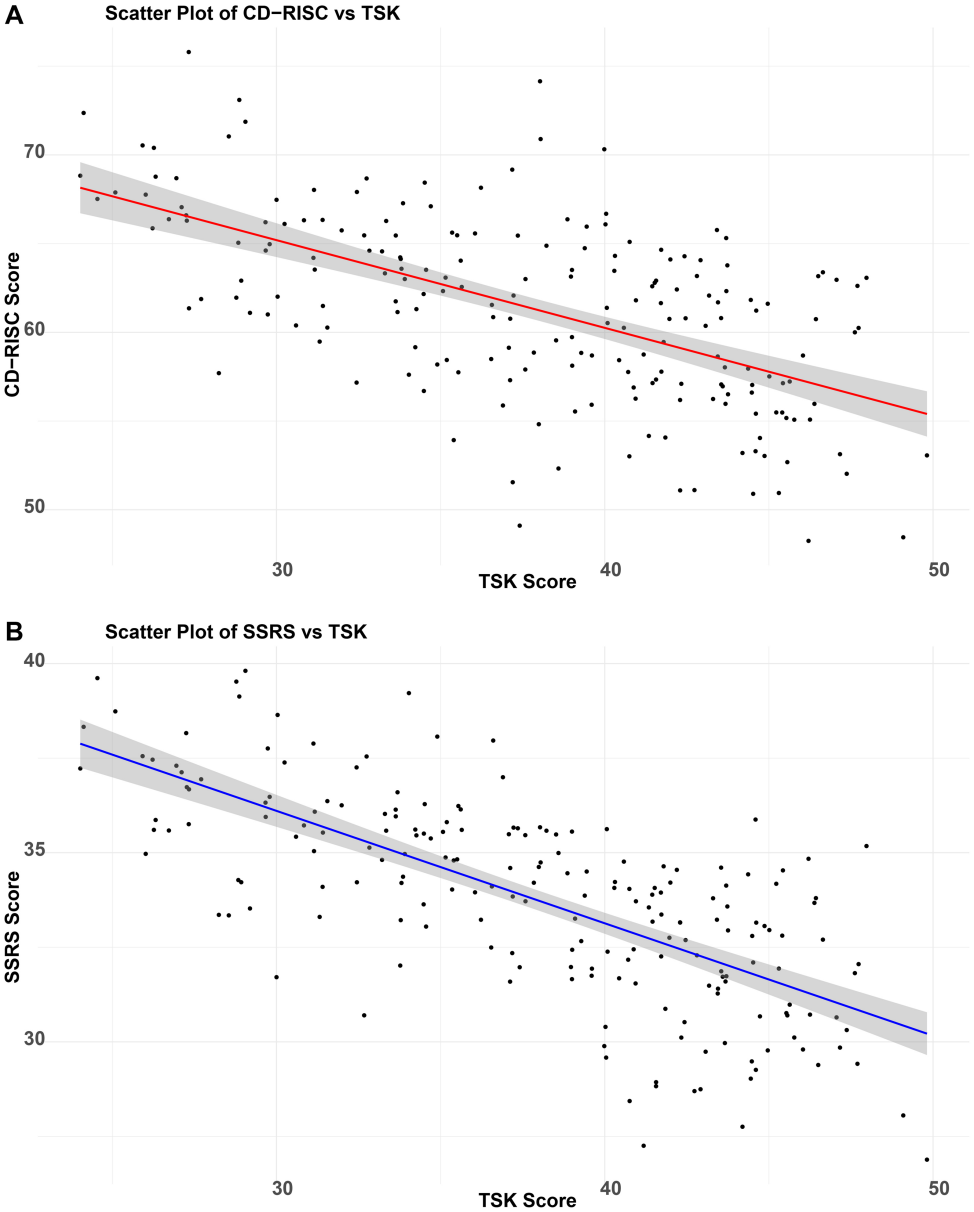
Impact of social support and psychological resilience on postoperative kinesiophobia in elderly patients with femoral neck fractures. **(A)** Correlation between SSRS and TSK scores in elderly femoral neck fracture patients; **(B)** Correlation between CD-RISC and TSK scores in elderly femoral neck fracture patients.

This study assessed levels of social support and psychological resilience in elderly patients following femoral neck fracture surgery. The mean SSRS score was 33.77 ± 2.64, and the mean CD-RISC score was 61.30 ± 5.25, indicating relatively low levels in the postoperative population. Correlation analysis showed a significant negative relationship between SSRS and TSK scores (r = −0.7057, *p <* 0.05), suggesting that higher social support was associated with milder kinesiophobia symptoms (Figure 3A). Similarly, CD-RISC scores were negatively correlated with TSK scores (*r* = −0.5912, *p <* 0.05), indicating that greater psychological resilience was also associated with lower levels of kinesiophobia (Figure 3B).

### Association between hospital stay duration, complication rates, and postoperative kinesiophobia

The average postoperative hospital stay was significantly longer in the kinesiophobia group (10.50 ± 2.10 days) compared to the non-kinesiophobia group (7.50 ± 1.80 days) (*t* = 12.34, *p <* 0.001), with a large effect size (d = 1.58, 95% CI: 1.41–1.75). The incidence of postoperative complications (e.g., infection, thrombosis) was also higher in the kinesiophobia group (6.67%) than in the non-kinesiophobia group (1.25%) (*χ*^2^ = 4.12, *p =* 0.042).

### Differences in patient satisfaction between kinesiophobia and non-kinesiophobia groups

The patient satisfaction survey (Appendix 2) revealed significantly lower overall satisfaction in the kinesiophobia group compared to the non-kinesiophobia group (68.2 ± 7.5 vs. 85.3 ± 5.1, *t* = 16.38, *p <* 0.001, d = 2.62). Domain-specific analysis demonstrated significant between-group differences across all assessed dimensions (*p <* 0.001), with particularly pronounced disparities in psychological support (2.9 ± 0.8 vs. 4.3 ± 0.7, d = 1.65) and functional recovery (2.8 ± 0.9 vs. 4.6 ± 0.7, d = 2.02). Notably, only 28% of kinesiophobia patients reported satisfaction with anxiety-relief interventions, compared to 82% in the non-kinesiophobia group (*χ*^2^ = 45.6, *p <* 0.001). Similarly, merely 25% of kinesiophobia patients expressed satisfaction with improvements in activity tolerance, significantly lower than the 85% satisfaction rate in controls. Additional significant differences emerged in pain management (3.2 ± 0.7 vs. 4.5 ± 0.6, d = 1.88), where only 32% of kinesiophobia patients considered analgesic regimens effective, and in social support (3.1 ± 0.7 vs. 4.5 ± 0.6, d = 1.58), particularly regarding access to family care resources (Table 6). Multivariate analysis identified poor overall satisfaction (OR = 1.402, 95% 1.214–2.153), inadequate pain management (OR = 1.289), and lack of psychological support (OR = 1.254) as independent risk factors for postoperative kinesiophobia (Table 7).

**Table 6:** Patient satisfaction survey.

Dimension	Kinesiophobia Group (*n* = 120)	No kinesiophobia Group (*n* = 80)	*t*/*χ^2^*	*p-value*
Satisfaction Score	68.2 ± 7.5	85.3 ± 5.1	t = 16.38	<0.001
Pain management	3.2 ± 0.7	4.5 ± 0.6	t = 12.74	<0.001
Rehabilitation guidance	3.6 ± 0.6	4.7 ± 0.5	t = 11.25	<0.001
Psychological support	2.9 ± 0.8	4.3 ± 0.7	t = 10.89	<0.001
Doctor-patient Communication	3.9 ± 0.6	4.8 ± 0.5	t = 9.67	<0.001
Social support	3.1 ± 0.7	4.5 ± 0.6	t = 10.12	<0.001
Recovery of Function	2.8 ± 0.9	4.6 ± 0.7	t = 13.45	<0.001

**Table 7:** Multivariate analysis of postoperative kinesiophobia risk factors (independent and non-significant factors).

Factor	Odds Ratio (OR)	95% Confidence Interval (CI)	*p*-value
Low overall satisfaction	1.402	1.214–2.153	<0.001
Inadequate pain management	1.289	1.107–1.502	<0.001
Lack of psychological support	1.254	1.058–1.487	<0.001
Insufficient rehabilitation guidance	1	0.800–1.200	0.902
Poor doctor-patient communication	1	0.800–1.200	0.898
Lack of social support	1	0.800–1.200	0.915
Impaired recovery of function	1	0.800–1.200	0.91

## Discussion

**Figure 4:**
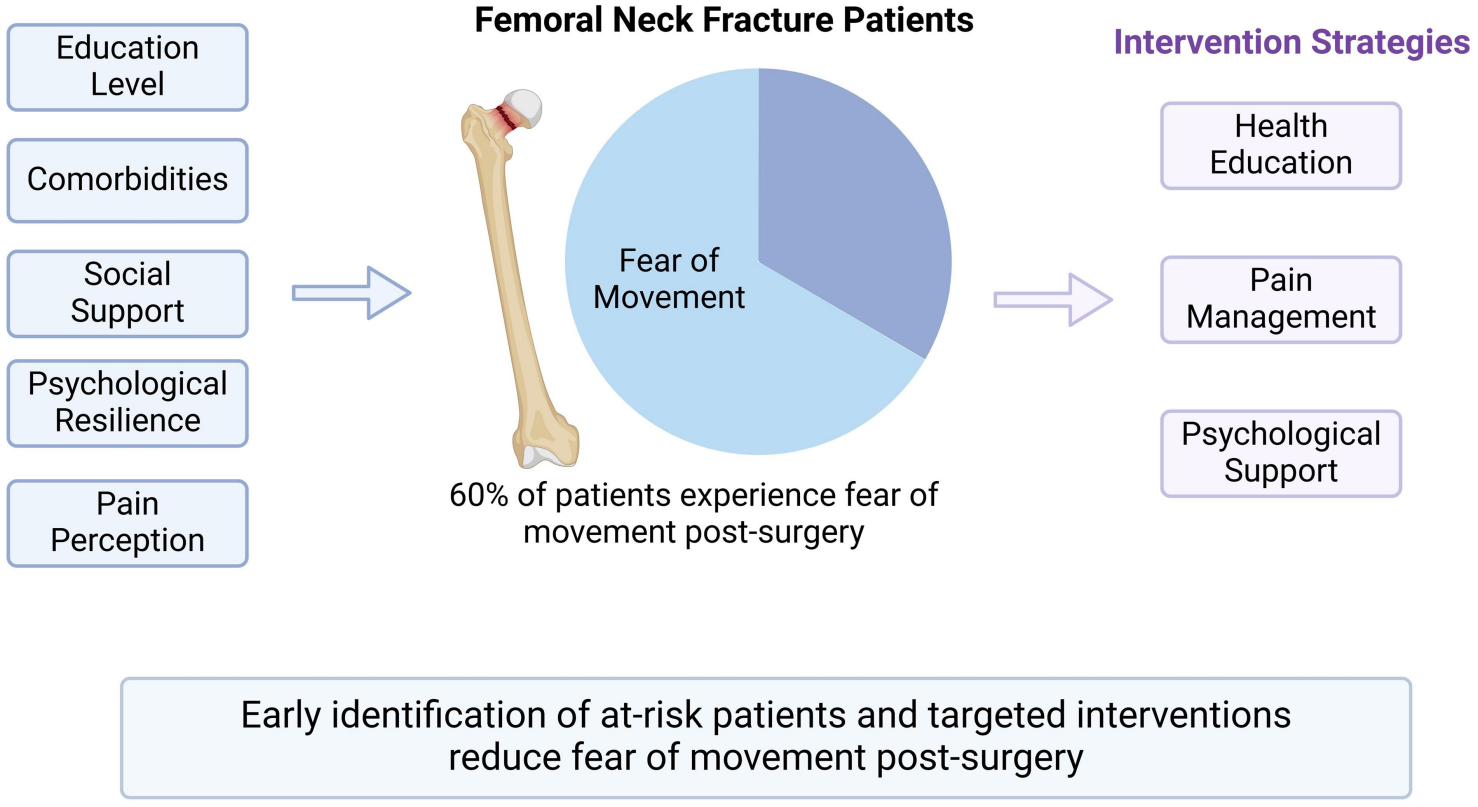
Factors influencing postoperative kinesiophobia and intervention strategies in elderly patients with femoral neck fractures.

An excessive fear of movement characterizes Kinesiophobia, often manifested as intense anxiety and avoidance behaviors related to physical activity. Its theoretical foundation is rooted in the fear-avoidance model, which posits that individuals who excessively worry about pain or injury may develop movement-related fear, ultimately leading to avoidant behavior (Wideman et al., 2013). Previous studies have indicated that postoperative patients with kinesiophobia tend to overestimate the risks associated with activity, which may lead to missed opportunities for optimal functional recovery (Bisson et al., 2022). This results in delayed rehabilitation, increased risks of complications such as muscle atrophy and thrombosis, and severely compromised long-term health outcomes (see Figure 4).

In this study, the incidence of postoperative kinesiophobia among elderly patients with femoral neck fractures reached 60%, indicating a high prevalence in this population. This is comparable to the reported 77% incidence in patients with knee osteoarthritis (Losciale et al., 2024) and the 56% rate observed in those undergoing minimally invasive surgery for chronic low back pain. The varying rates across populations may be attributed to differences in disease type, surgical method, educational level, and social support. Our analysis also demonstrated a significant association between kinesiophobia and prolonged hospital stay as well as an increased risk of postoperative complications. Previous research has shown that kinesiophobia may extend hospitalization duration by an average of 30% (Li et al., 2019). A meta-analysis reported that for each standard deviation increase in kinesiophobia scores, the risk of developing deep vein thrombosis rose by 1.8 times (OR = 1.82, 95% CI: 1.24–2.68) (Keddie et al., 2018), while a prospective study indicated that patients with kinesiophobia were 2.3 times more likely to be readmitted within 3 months postoperatively (HR = 2.31, *p =* 0.003) (Gruneir et al., 2016). Collectively, these findings support the psychophysiological vicious cycle theory, wherein fear-induced avoidance behaviors contribute to muscle deconditioning and hemodynamic disturbances, thereby exacerbating poor surgical outcomes.

Multiple influencing factors were identified about postoperative kinesiophobia, including educational attainment, comorbidity burden, pain severity, self-efficacy, and psychosocial resources. Higher educational levels were associated with a lower risk of kinesiophobia, consistent with findings from studies on total knee arthroplasty patients (Du et al., 2025). Patients with limited education often face barriers in understanding rehabilitation information and engaging actively in recovery. In our study, only 35% of patients with low educational backgrounds actively sought rehabilitation guidance, reflecting both cognitive and behavioral disadvantages. This population also demonstrated lower perioperative compliance (Vu et al., 2024; Norekvål et al., 2020; Rolls et al., 2017). A comorbidity count of ≥2 significantly increased the risk of kinesiophobia (Zeng et al., 2024), likely due to elevated recovery challenges and psychological stress (Helminen et al., 2020). Moreover, higher pain scores (NRS) were strongly associated with kinesiophobia, aligning with the fear-avoidance framework (Wideman et al., 2013). Pain-related fear can disrupt gait and biomechanical stability, intensifying avoidance behavior. Individuals with low self-efficacy are particularly vulnerable to catastrophic interpretations of pain, perpetuating the fear-avoidance cycle (Brindisino et al., 2023; Brown et al., 2020; Terradas-Monllor et al., 2024).

Psychosocial factors also play a crucial role in the development of postoperative kinesiophobia. In this study, lower scores on the SSRS and the CD-RISC were significantly associated with increased kinesiophobia, and both were negatively correlated with TSK scores (Greenberg et al., 2021). These findings are consistent with prior evidence that enhanced social support alleviates postoperative anxiety and fear (Ji et al., 2022; Li et al., 2024). Self-efficacy, as a core psychological resource in regulating behavioral responses, was further confirmed as a protective factor in our study. Patients with lower self-efficacy scores had significantly higher rates of kinesiophobia (OR = 1.242), corroborating multiple prior findings (Cao et al., 2025; Zhang et al., 2022; Zelle et al., 2016; Westerdahl et al., 2024; Jakobsson et al., 2023). These results offer a theoretical foundation for identifying high-risk individuals and tailoring early interventions. Future research should further investigate the interactions between pain sensitivity, pain duration, and self-efficacy to inform precise and targeted intervention strategies.

Based on these mechanisms and findings, this study proposes several rehabilitation-based intervention strategies. For patients with low educational attainment, we recommend large-font illustrated brochures and animated videos demonstrating safe activity postures, supplemented by caregiver-assisted education. Screening using the simplified TSK-11 scale should be conducted within 24 h postoperatively, with high-risk individuals receiving graded exposure therapy. At the community level, the FRAIL scale can be used to assess home environment risks (e.g., anti-slip flooring, handrail installation), with rehabilitation professionals providing biweekly home visits for high-risk families. Pain management should also be prioritized as a core measure in the prevention of kinesiophobia. Rehabilitation strategies must center around functional recovery and incorporate patients’ educational levels, pain intensity, self-efficacy, social support, and resilience to develop staged and individualized care plans, thereby enhancing compliance and reducing long-term disability risk.

This study has several limitations. First, the single-center convenience sampling, conducted in a tertiary hospital, may have introduced selection bias and limits generalizability. Second, although the sample size met statistical requirements, the high prevalence of kinesiophobia suggests that a larger cohort would enhance external validity. Third, despite controlling for key confounders, unmeasured variables such as socioeconomic status and quality of home care may have influenced the accuracy of our assessment of social support. Fourth, the TSK was administered only once on postoperative day 3, which restricts understanding of the dynamic evolution of kinesiophobia symptoms.

Future research should consider the following directions: (1) establish multicenter prospective cohorts to improve representativeness; (2) apply stratified random sampling to include different healthcare levels and community-dwelling populations for greater ecological validity; (3) incorporate interventional studies using assessor blinding, third-party imaging assessments of function, and longitudinal symptom monitoring; (4) use WHO-SES scales to quantify socioeconomic status as a potential mediator or moderator; and (5) conduct multi-timepoint evaluations to map the trajectory of kinesiophobia and support precision intervention.

This study confirms a high incidence of postoperative kinesiophobia among elderly patients with femoral neck fractures, with strong associations observed across educational background, comorbidities, pain levels, social support, psychological resilience, and self-efficacy. Early identification of high-risk populations and the implementation of region-specific health education and personalized rehabilitation interventions may reduce the incidence of postoperative kinesiophobia, improve functional recovery, and ultimately enhance long-term patient independence and outcomes.

## Conclusion

This study identified the multidimensional determinants of postoperative kinesiophobia in elderly patients with femoral neck fractures and established a core high-risk profile characterized by the triad of low educational attainment, multimorbidity, and limited psychological resources. Grounded in the Fear-Avoidance Model and Self-Determination Theory, we propose a three-tier translational intervention pathway: rapid risk stratification within 24 h postoperatively based on education and comorbidity indices; community nurse-led dual-module training targeting social support and psychological resilience; and integration of kinesiophobia management into the ERAS pathway for geriatric hip fractures to establish a closed-loop continuum from acute care to community rehabilitation.

Given the modifiability of multiple risk factors associated with kinesiophobia, we recommend the systematic incorporation of psychosocial assessments and health education into the perioperative care process to enhance rehabilitation adherence, reduce long-term disability risk, and promote individualized, precision-oriented management of geriatric fracture recovery. Future research should focus on the dynamic trajectories of kinesiophobia, the development of elderly-friendly digital intervention tools, and the long-term health economic outcomes of such strategies.

## Data Availability

The original contributions presented in the study are included in the article/Supplementary material, further inquiries can be directed to the corresponding author.
