# Supplementary material for: Postoperative kinesiophobia in elderly patients with femoral neck fractures: a prospective study of psychological and social determinants
Source: Front Psychol. 2025 Oct 31;16:1622585. doi: 10.3389/fpsyg.2025.1622585 (PMC12615442; doi:10.3389/fpsyg.2025.1622585)
Supplement: Supplementary file 1 [file Supplementary_file_1.docx]

**General Information Questionnaire**

Thank you for participating in this study. Please answer the following questions based on your basic information. All data will be used solely for this research, and your responses will help us better understand patient conditions and improve treatment processes.

**1. Gender:**

- Male

- Female

**2. Age (actual age):** _________ years

**3. Body Mass Index (BMI) (actual value):** _________ kg/m²

**4. Residence:**

- Urban

- Rural

**5. Marital Status:**

- Married

- Unmarried/Widowed

**6. Education Level:**

- High school or below

- College or abov

**7. Smoking History:**

- Yes

- No

**8. Alcohol Consumption History:**

- Yes

- No

**9. Number of Comorbidities:**

- 0-2

- >2

**10. Diagnosis:**

- Right femoral neck fracture

- Left femoral neck fracture

**11. Health Insurance Type:**

- Employee medical insurance

- New Rural Cooperative Medical Scheme (NRCMS)

**12. Time from Fracture to Surgery (actual duration): _________ days**

**13. Anesthesia Method:**

- Spinal anesthesia

- General anesthesia
