# Supplementary material for: Postoperative kinesiophobia in elderly patients with femoral neck fractures: a prospective study of psychological and social determinants
Source: Front Psychol. 2025 Oct 31;16:1622585. doi: 10.3389/fpsyg.2025.1622585 (PMC12615442; doi:10.3389/fpsyg.2025.1622585)
Supplement: Supplementary file 2 [file Supplementary_file_2.docx]

**Postoperative Satisfaction Questionnaire for Elderly Patients with Femoral Neck Fractures**

Thank you for participating in this study. Please answer the following questions based on your true postoperative experience. Your responses will help us improve future rehabilitation treatments. Select the option that best matches your current feelings for each question.

**1. Overall satisfaction with treatment:**

- **0–20** = Very dissatisfied
- **20–40** = Dissatisfied
- **40–60** = Neutral
- **60–80** = Satisfied
- **80–100** = Very satisfied

**2. Satisfaction with postoperative pain management (e.g., analgesic effect, pain relief measures):**

- **1** = Very dissatisfied
- **2** = Dissatisfied
- **3** = Neutral
- **4** = Satisfied
- **5** = Very satisfied

**3. Satisfaction with postoperative rehabilitation guidance (e.g., recovery advice, exercise instructions):**

- **1** = Very dissatisfied
- **2** = Dissatisfied
- **3** = Neutral
- **4** = Satisfied
- **5** = Very satisfied

**4. Satisfaction with postoperative psychological support (e.g., emotional counseling and care from doctors/nurses):**

- **1** = Very dissatisfied
- **2** = Dissatisfied
- **3** = Neutral
- **4** = Satisfied
- **5** = Very satisfied

**5. Satisfaction with postoperative doctor-patient communication (e.g., adequacy of communication, clarity of information):**

- **1** = Very dissatisfied
- **2** = Dissatisfied
- **3** = Neutral
- **4** = Satisfied
- **5** = Very satisfied

**6. Satisfaction with postoperative social support (e.g., family care, friend assistance, social resources):**

- **1** = Very dissatisfied
- **2** = Dissatisfied
- **3** = Neutral
- **4** = Satisfied
- **5** = Very satisfied

**7. Satisfaction with postoperative functional recovery (e.g., regained mobility, ability to perform daily activities):**

- **1** = Very dissatisfied
- **2** = Dissatisfied
- **3** = Neutral
- **4** = Satisfied
- **5** = Very satisfied

### ****Data Collection Instructions****

1. Questionnaire completion time: Patients should complete the questionnaire on postoperative Day 3 when their condition is stable.
2. Data collection method: Researchers will provide each patient with a paper or electronic version of the questionnaire, ensuring it is completed in a quiet environment.
3. Privacy protection: All patient information will remain strictly confidential, and data will be used only for statistical analysis in this study.
4. Statistical analysis: Questionnaire data will be analyzed to compare satisfaction differences between the kinesiophobia group and non-kinesiophobia group across various dimensions.
